# Evidence of latency reshapes our understanding of Ebola virus reservoir dynamics

**DOI:** 10.1101/2025.10.17.683141

**Published:** 2025-10-18

**Authors:** John T. McCrone, Guy Baele, Ifeanyi F. Omah, Eddy Kinganda-Lusamaki, Joseph A. Brew, Luiz M. Carvalho, Gytis Dudas, Placide Mbala-Kingebeni, Marc A. Suchard, Andrew Rambaut

**Affiliations:** 1 Vaccine and Infectious Disease Division, Fred Hutchinson Cancer Center, Seattle, Washington, USA; 2 Department of Microbiology, Immunology and Transplantation, Rega Institute, KU Leuven, Leuven, Belgium; 3 Institute of Ecology and Evolution, University of Edinburgh, Edinburgh, UK; 4 Institut National de Recherché Biomedicale, University of Kinshasa, Kinshasa, Democratic Republic of the Congo; 5 TransVIHMI, Université de Montpellier, Montpellier, France; 6 Department of Biomedical Informatics, Harvard Medical School, Boston, MA, USA; 7 School of Applied Mathematics, Getulio Vargas Foundation (FGV), Rio de Janeiro, Brazil; 8 Institute of Biotechnology, Life Sciences Centre, Vilnius University, Vilnius, Lithuania; 9 South African National Bioinformatics Institute, University of the Western Cape, South Africa; 10 Department of Biostatistics, Fielding School of Public Health, University of California, Los Angeles, CA, USA; 11 Department of Biomathematics, David Geffen School of Medicine, University of California, Los Angeles, CA, USA; 12 Department of Human Genetics, David Geffen School of Medicine, University of California, Los Angeles, CA, USA

## Abstract

Ebola virus (EBOV) has caused severe outbreaks of haemorrhagic fever in Central and West Africa since the first observed zoonotic epidemic in the late 1970s. While recent outbreaks have revealed much about the epidemiological dynamics that sustain human-to-human transmission, the mechanisms by which the virus persists between outbreaks are unknown. Previously, phylogenetic approaches have been used to characterise the EBOV reservoir from the evolutionary relationships among observed human outbreaks. We here employ a novel phylogenetic latency model – inspired by recent observations of extreme EBOV evolutionary rate heterogeneity in humans – to characterise the natural history of EBOV and by extension its reservoir. We find the prevailing model of EBOV reservoir dynamics is deficient, and the long-term EBOV evolutionary rate is slower than previously believed. The hypothesis that EBOV diversity dates back to a bottleneck event just prior to the first human outbreak is not supported by the data. Further, our results suggest that EBOV undergoes extended periods of quiescence in the reservoir, similar to that observed in a small fraction of human infections. These findings have significant implications for understanding the source of EBOV outbreaks, characterising the EBOV reservoir, and uncovering the factors that contribute to EBOV outbreaks in humans.

## Introduction

Ebola virus (EBOV) is a filovirus of the genus *Ebola* which causes Ebola virus disease (EVD), a severe haemorrhagic fever in humans. The first recorded outbreak of EBOV occurred in 1976 near Yambuku, in the north of what is now the Democratic Republic of the Congo (DRC) (1). The epidemic spread through the Yambuku Mission Hospital resulting in 318 cases of EVD and 280 deaths. Because much of the transmission was nosocomial and the disease was unknown to the people in the region, epidemiologists suspected the virus was imported into the clinic by a traveller from Sudan, where a separate outbreak of haemorrhagic fever had also been reported. However, the WHO bulletin report from the outbreak noted that EBOV may be endemic to the area and reside in an unknown reservoir due to the discovery of anti-EBOV glycoprotein (GP) antibodies in five individuals who were not ill and did not have a connection with the recorded outbreak (1). Later, it was shown that the Sudan outbreak was caused by a separate filovirus – Ebola virus Sudan – further supporting a separate zoonotic origin of the outbreak in Yambuku (2). Since EBOV was first isolated in 1976, there have been at least 18 detected outbreaks sparked by independent zoonoses. It is now widely accepted that EBOV is endemic in the wildlife of Central Africa. However, the exact reservoir of EBOV remains uncharacterised.

EBOV is thought to be maintained among bat populations in Central Africa, based largely on observations that Marburg virus, a related filovirus, transmits among fruit bats (3, 4). Field work supports this hypothesis. Small fragments (~265bp) of EBOV genomes as well as anti-Ebola GP antibodies were isolated from fruit bats sampled near human outbreaks along the border of the Republic of the Congo and Gabon in the early 2000s (5). Additionally, in 2019 EcoHealthAlliance sequenced a fragment of the virus from a greater long-fingered bat (*Miniopterus inflatus*) in Liberia (6–8).

But the reservoir is likely more complex than one species or population. Partial sequences isolated from gorilla, chimpanzee, and duiker carcasses associated with human outbreaks in Central Africa, and serology from non-human primates suggest multiple species act as intermediate hosts (9–11). The degree to which any particular species contributes to the long-term reservoir is unknown. Neither live virus, nor whole genome has been isolated from a bat population, and concentrated sampling efforts near recent outbreaks have failed to find an active reservoir (12). Much of what is known about the EBOV reservoir has been derived from evolutionary analyses, which impute population dynamics from the ancestral relationships among zoonotic spill-overs (11, 13, 14). In the absence of any EBOV genome sequences from infected reservoir species, we are observing the evolutionary history of EBOV in non-human animals entirely from outbreaks and zoonotic infections in humans.

**Figure 1:**
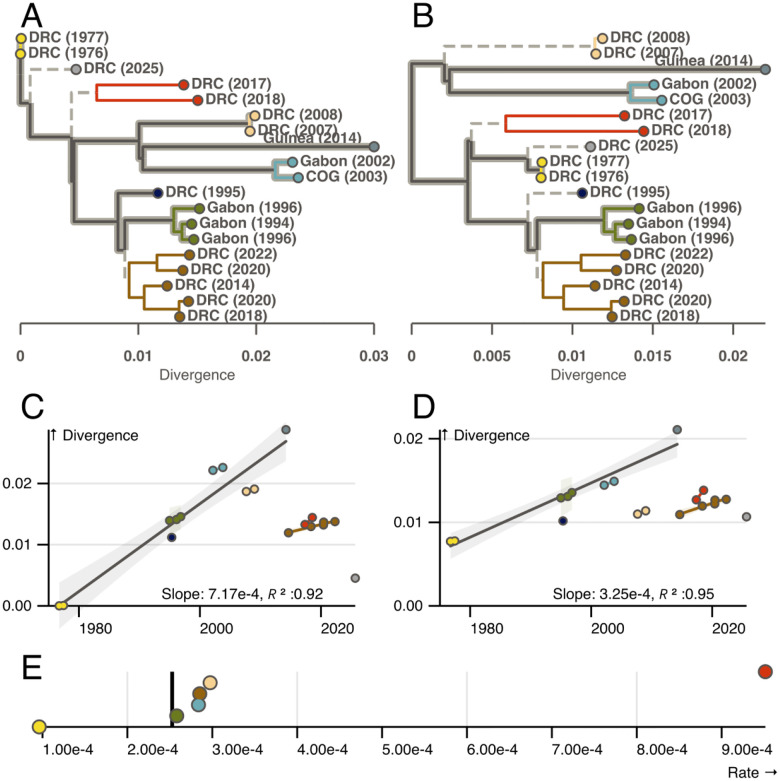
Root-to-tip exploration of rate heterogeneity in the EBOV phylogeny. A & C) The EBOV phylogeny rooted at the accepted 1970s outbreak position and corresponding root-to-tip divergence plot. B & D) Similar to A and C but with the proposed rooting. In all figures tips are coloured by outbreak cluster (Supplemental Figure S1A) and labeled by country and year of outbreak. Branches in A and B that are highlighted in grey connect tips used in the regressions in C and D. Smaller, coloured regression connect tips belonging to the same cluster. E) The maximum-likelihood evolutionary rate estimated for each cluster independently. The black line represents the evolutionary rate estimate when all clusters share the same rate.

It has long been suggested that EBOV diversity dates back to the 1970s outbreaks of Yambuku (1976) and nearby Bonduni (1977) (13–17). This prevailing view relies heavily on a temporal rooting of the EBOV phylogeny estimated from isolates sampled between the 1976 outbreak and the West African outbreak of 2013. Phylogeographic models based on this origin found EBOV exhibited wave-like spread out of the northern DRC, and proposed an active expanding epidemic within the unobserved reservoir (14). The location of the 2013 West African outbreak drew this geographic model into question, and led some to propose a new root position based on outgroup rooting with other *Ebola* species. This analysis found the West African outbreak as an outgroup to all other EBOV lineages (18). However, outgroup rooting with divergent species is unreliable (19), and while the epidemic in West Africa was a geographic outlier (Supplemental Figure S1A), its divergence from the 1976 Yambuku outbreak is consistent with previous estimates of the virus’s evolutionary rate (Figure 1A) (20). The prevailing model, based on a temporal rooting, has persisted to this day.

There have been eight sequenced zoonotic outbreaks of EBOV since 2013 (21–25) (Supplemental Table S1). The most recent occurred in August, 2025 (26). All have been less diverged than expected given the accepted model (Figure 1), leading some to hypothesize that EBOV may have entered a new reservoir population with slower replication dynamics (21). However, recent observations of EBOV rate heterogeneity in humans offer a different perspective. There are now multiple, well-documented cases in which EBOV has persisted for extended periods of time (weeks to years) in a recovered individual, before sparking a secondary transmission chain (27–30). The maximum length of EBOV persistence in humans is unknown. The most extreme case to date lasted six years (30), suggesting EBOV may persist in hosts for much longer than initially thought. Genomic sequencing has revealed that fewer mutations accumulate during these long periods of persistence than expected given the EBOV evolutionary rate (28, 30). How infectious EBOV persists in a host for long periods of time without mutating (and by extension replicating) is unknown. The epidemiology of these well-documented events implies it must.

Observations of the persistence phenomenon in survivors of acute EVD suggest that, within those individuals, persistence is a two-state process with replication (and thus mutation) only occurring during the initial infection and at the point of transmission (either before or after). Recrudescence of EVD — the re-emergence of replicating virus — after a period of remission has been observed (31), but does not seem to be required as most secondary transmission chains have begun through sexual transmission without re-establishment of replication in the survivor. For the Guinea 2021 outbreak, linked to the 2013–2016 epidemic by a period of over six years, the index case suffered acute, and fatal, EVD but was not known to have been infected previously nor to have had contacts who were survivors (30). In this case, the virus exhibited almost no genetic divergence from the isolates taken during the epidemic suggesting most of the 6 year period was spent in a non-replicating, latent, state.

We hypothesize that the decreased divergence associated with recent zoonotic events results from a latent process in the EBOV reservoir similar to that observed in persistent human infections, and that accounting for these dynamics could reconcile recent observations with the long-held model of EBOV’s evolutionary past. Here, we have developed a model of the virus’s evolutionary rate that accounts for periods of latency, and applied it to a dataset representing EBOV evolution in the reservoir. Our findings suggest EBOV latency is not unique to human infections, but rather has occurred throughout the natural history of EBOV. Furthermore, accounting for these dynamics draws the prevailing model of long-term EBOV evolution into question and redefines our expectations for the timing and location of future outbreaks.

## Results

We collected a representative set of Ebola virus genomes, taken from 19 plausibly independent zoonotic spill-over events (Table S2). The genomes included in this study were sequenced from human subjects, but because we restrict our sampling to one genome per outbreak and prioritize early samples, our phylogeny is largely shaped by the evolutionary dynamics in the reservoir.

Restricting our dataset to genomes from the eleven recorded zoonotic outbreaks between 1976 and 2013, and rooting near the 1976 Yambuku outbreak recovers the accepted model of EBOV evolution and yields a root-to-tip regression with a high correlation coefficient and an R^2^ of 0.92 (Figure 1A & C). This rooting also produces an evolutionary rate of 7.15 × 10^−4^ substitutions per site per year (s/s/y), not dissimilar to the 1.3 × 10^−3^ s/s/y observed during human outbreaks (32).

However, as Lam *et al*. (21) and later Mbala-Kingebeni *et al*. (33) observed, this visually appealing linear relationship breaks down with the 2014 DRC and subsequent outbreaks (Figure 1C). Attempts by us (34) and others (33) to account for the limited divergence of recent outbreaks have required topological constraints and/or informative priors on the molecular clock rate in addition to *a priori* assigned local clock models in order to recover a root near the original 1976 Yambuku outbreak. The necessity of these informative priors in addition to *a priori* assigned local clocks suggests the temporal signal in the first 11 outbreaks is not as strong as initially thought.

### Evidence for an alternative root position

It has been noted elsewhere (33) that the EBOV phylogeny is made up of several clades of outbreaks that cluster by time and location (denoted by colour in Figure 1 and S1). We observed that the root-to-tip regression based on a root near the 1976 outbreak is driven by the long branches that connect these clusters (Figure 1A and C).

However, there seems to be an alternative regression within each cluster (coloured clades in Figure 1), that differs consistently from this trend. The rate implied by these independent regression slopes is slower than that expected if EBOV diversity descends from a common ancestor near the 1976 Yambuku outbreak (Figure 1C).

We estimated the evolutionary rate of each cluster in Figure 1 by maximum-likelihood and found remarkably similar rates across many clusters (Figure 1E). We observed a slower rate of evolution among the 1970s outbreaks, although our power to estimate such a rate in the absence of many mutations is low. We also found the clade representing the 2017 and 2018 Northern DRC outbreaks may exhibit a higher rate of evolution than other clades. While this is consistent with previous results (33), we note that this clade is characterised by long external branches which corresponds to a high variance in the estimated rate.

We next explored possible partitions of the data that would allow for similar within- and between-cluster evolutionary rates. There is no rooting that does not require root-to-tip outliers with less divergence than their date would suggest. However, if we root the EBOV phylogeny as in Figure 1B, there is a subset of branches that pass through the root, connect several outbreak clusters, and imply a similar between- and within- cluster evolutionary rate.

We found statistical support for clock-like evolution with an identifiable rate in the subtree of eight outbreaks highlighted in Figure 1B when rooted at this proposed root (35). The proposed root position had the highest log-likelihood of all tested rootings (“Proposed” in Table 1), but temporal signal was also observed when the tree was midpoint rooted with the 2013 West African outbreak as an outgroup. Interestingly, we found no evidence for clock-like evolution in this dataset with the accepted root position near the 1976 Yambuku outbreak. These signals were maintained in the 1976–2013 dataset (“Classic temporal set”) as well as in the full dataset of 19 samples when local clocks were applied to branches subtending the remaining clades (dashed branches in Figure 1B; Table 1). Our findings suggest that while the current model of EBOV is likely based on a misguided interpretation of root-to-tip correlations, clock-like evolution with periodic rate heterogeneity is a plausible explanation for EBOV evolutionary dynamics.

### A mechanistic rate model discovers the same evolutionary dynamics

We sought to determine if our proposed model for EBOV evolution is consistent with latency in the reservoir population. To this end, we developed a state-dependent evolutionary rate model in which lineages transition between latent and replicating states (see Methods). When in the replicating state, substitutions accumulate according to an underlying evolutionary rate; however, the rate is zero whilst in the latent state. We constrain our model such that all nodes are in the replicating state. External nodes represent sampled, active infections and internal nodes represent bifurcation events - both processes that require that the virus was replicating. Because of this constraint, latency is less likely to be observed on branches that span short periods of time, such as those seen within outbreak clusters, and more likely on the longer branches that connect outbreaks.

We initially focused on the 18 outbreaks identified in Central Africa, based on the assumption that these may be connected by a contiguous reservoir population that excludes the geographically distant 2013 West African outbreak. We found the proposed root had the highest posterior support (74.4%) with the remaining support split between trees with the 2002/2003 Gabon clade (blue) or the 2007/2008 DRC clade (peach) as outgroups. (Supplemental Figure S2) This analysis resulted in a mean root age of 1931 (95% HPD [1892, 1956]) which corresponded to a mean replicating evolutionary rate of 2.34×10^−4^ s/s/y (95% HPD [1.43×10^−4^, 3.19×10^−4^]). We estimated a mean latent-replicating transition rate of 0.07 (95% HPD [0.03, 0.10]) transitions per year with a mean bias of 0.70 (95% HPD [0.429, 0.951]) towards entering the latent state. These parameters result in a median of 6 branches with latency (95% HPD [5, 9]). The root position, root age, and evolutionary rate are all consistent with our exploration above. Further, the five branches observed to be outliers in the root-to-tip plots are the only branches with a greater than 50% posterior probability of at least one latent period.

Despite being a geographic outlier, 2013 West African outbreak is not a temporal outlier (Figure 1D). The large number of substitutions that distinguish this outbreak from the other EBOV isolates are consistent with our estimated rooting and evolutionary rate (Figure 1) and suggests little to no latency has occurred on this branch. We wanted to explore whether or not a branch of this length is consistent with our estimates of latency. If we assume this outbreak diverges from the rest of EBOV near the root estimated above, its expected branch length would be 83 years long (95% HPD [58, 122]) which corresponds to a 5.61% probability (0.84%, 18.50%) of no latency.

We found our model was largely robust to inclusion of this possible outlier, with the exception of increased uncertainty in the EBOV root placement. The mean root age of the entire dataset was 1922 (95% HPD [1884, 1954]) which corresponded to a replicating evolutionary rate of 2.37×10^−4^ substitutions per site per year (95% HPD [1.51×10^−4^, 3.12×10^−4^]) and a median of 7 branches with latency (95% HPD [5, 10]). The underlying evolutionary rate is consistent with that expected from the root-to-tip analysis and maximum-likelihood estimates above, but we find less support for the proposed root position (19.7% posterior support). Instead the models favours a root with the West African outbreak as an outgroup (27.4%). The majority of the remaining posterior support was again split between trees with the 2002/2003 Gabon clade (blue) or the 2007/2008 DRC clade (peach) as outgroups (Supplemental Figure S3).

Recent work by Gao *et al*. (36) has shown that posterior distributions can be disproportionately affected by the inclusion of a small number of taxa. Our estimate of the EBOV root position is largely affected by the inclusion of the 2013 West African outbreak. If this outbreak represents the true EBOV outgroup, the root age is likely much older than that estimated by our model, as the duration of latency on this branch (and its sibling) is only informed by the parameter estimates from other parts of the tree. However, we are able to estimate the root of the remaining 18 outbreaks with reasonable confidence. While the rootings in Supplemental Figure S3 represent 4 distinct topologies, they all lie near our proposed root, where several long branches diverge in rapid succession (Supplemental Figure S1B). The uncertainty in the root position is defined entirely by uncertainty in the order of these events, leading us to conclude the proposed root is the most probable EBOV root position.

### Consequences of latency for future outbreaks

**Figure 2:**
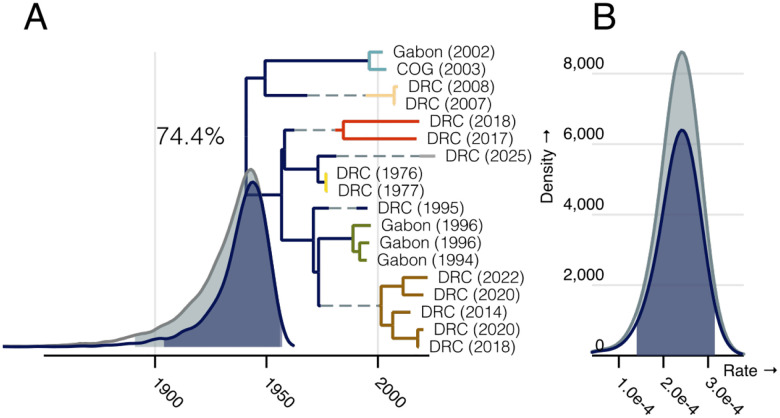
Posterior distributions of latency model parameter estimates when the West African outbreak is excluded. A) A maximum clade credibility (MCC) tree with the posterior probability of the root position noted near the root. Clades are coloured as previously. The root age distribution of the entire analysis is shown in grey with the contribution of the displayed root highlighted in blue. The mean duration of latency (conditioned on there being at least one period of latency) is shown as a dashed line on branches with a posterior probability of any latency greater than 50%. B) The posterior distribution of the evolutionary rate during replication, again shaded by total (grey) and root-specific conditioning (blue). In all posterior distribution plots 95% HPD intervals are shaded.

**Figure 4:**
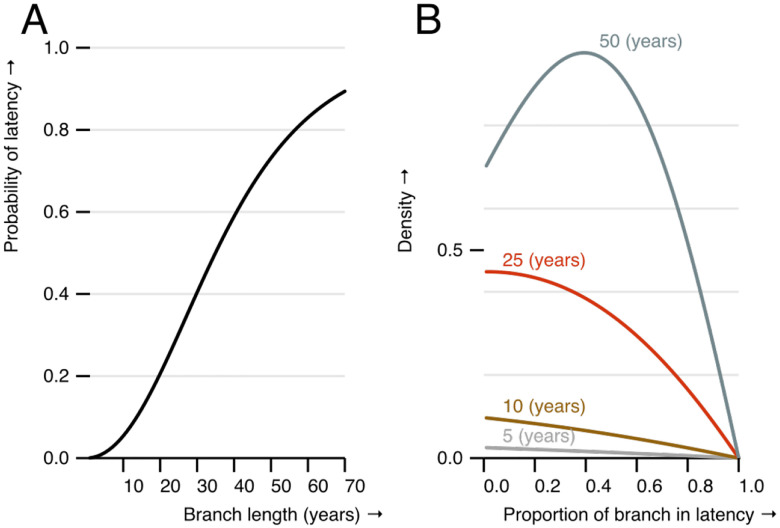
The expected contribution of latency to future outbreaks based on model estimates. A) The probability of some latency as a function of branch length. B) The distribution of the proportion of time spent in latency provided there is some latency on branches of different length (noted text near each line).

Our results suggest a significant number of EBOV outbreaks have derived from once latent lineages, and that latency may be an evolutionary mechanism by which EBOV persists in the reservoir. We were interested in the contribution of latency to future EBOV outbreaks, and plotted a cumulative distribution of there being at least one latent period as a function of branch length using the parameters estimated by our model (Figure 4A). We excluded the West African outbreak in these parameter estimates and relied on the analysis presented in Figure 2 on the basis that the West African outbreak seems to have originated from a separate process than that acting in Central Africa. Our results suggest a branch length of 35 years has a 50% chance of exhibiting latency, and the expected proportion of time spent latent for such a branch is 0.4 or roughly 14 years. As highlighted in Figure 4B, we have little precision in our estimate of how much latency to expect provided there is latency on a branch.

Our model above, finds latency on the branch subtending the recent EBOV outbreak in Kasai, DRC. This is consistent with the limited number of mutations (96) that have accumulated over the 49 years that have passed between this outbreak its closest relative — the 1976 Yambuku outbreak. We wondered if our model estimates could be used to predict the contribution of latency to this outbreak prior to genome sequencing. Fitting the latent model to the Central African dataset without the Kasaï outbreak resulted in similar parameter estimates to those reported above: mean transition rate of 0.07 (95% HPD [0.03, 0.10]) per year with a mean bias of 0.67 (95% HPD [0.373, 0.947]) towards entering the latent state. These estimates predict the Kasaï outbreak has an expected probability of 61.8% of at least one latent period. As expected, conditional on there being latency, the model provides a wide range of possible durations. The expected time spent latent is 20 years (95% quantiles [6, 48]), which corresponds to an evolutionary rate of 1.35×10^−4^ s/s/y (95% quantiles [3.20×10^−5^, 2.51×10^−4^]) or roughly 134 mutations (95% quantiles [28, 218]). The observed 96 mutations falls within this range; however we expect a more detailed model that accounts for the fragmented nature of the EBOV reservoir may better predict which clades are likely replicating, and which may be expected to persist only through latency.

## Discussion

An accurate model of EBOV evolution in the reservoir has important consequences for how we classify observed outbreaks and evaluate the risk of future epidemics. Following the Guinea outbreak of 2021, which was linked to the 2013 West African outbreak, it was proposed that decreased rates of evolution could be used to identify human-derived outbreaks (37). In the initial phylogenetic analysis of the Equateur/2020 outbreak in the DRC one genome was found to cluster near the base of the Equateur/2018 outbreak (23). Because of this placement, and the lack of rapid evolution, the sample was thought to represent a persistent human case from the Equateur/2018 outbreak. However, there was no epidemiological evidence for this noted at the time, and this sequence’s divergence is consistent with our modified estimate of Ebola reservoir dynamics (Note the two DRC (2020) isolates in Figure 1). Given our updated model, this sample likely represents an independent spillover event from a large, diverse reservoir active in Equateur province, DRC, between 2018–2022, and not a persistent human case.

Our updated estimate of the evolutionary rate of EBOV in the reservoir has additional implications for our understanding of EBOV evolutionary dynamics. The evolutionary rate of the 2013 West African outbreak was initially observed to be slightly higher than the prevailing estimate of the reservoir rate (38, 39). This is expected as decreased purifying selection is known to elevate evolutionary rate estimates taken during acute outbreaks (40). These estimates are consistent with a single EBOV evolutionary rate in humans and the reservoir. However, our estimated EBOV evolutionary rate is less than half of previous estimates, suggesting EBOV evolution may evolve by host-specific evolutionary processes (i.e. with elevated rates in human populations).

Given our finding that latency is wide-spread in EBOV evolution, it may be tempting to conclude that many EBOV outbreaks have resulted from persistently infected survivors of previous, unobserved outbreaks. Perhaps humans make up a significant proportion of the reservoir. We believe this to be implausible. Persistence in humans, although observed, is exceedingly rare, and only associated with the largest outbreaks and epidemics. Such an EBOV reservoir would require large, unobserved human epidemics. Additionally, our analysis identifies latency on several internal branches, precluding the possibility that these branches represent persistent human infections.

Our proposed root yields an older date for the common ancestor EBOV diversity and has important implications for understanding the geographic distribution of the virus. The older root provides more time for EBOV to have expanded into its wide geographic range, while maintaining the possibility of wave-like spread in a subclade of the tree. Our model also suggests EBOV diversity predates the first observed outbreaks in the 1970s, and does not require EBOV to have emerged from a genetic bottleneck just prior to the 1976 Yambuku outbreak. Although seemingly supported by the root-to-tip model at the time, this bottleneck and subsequent rapid expansion across the continent defied ready explanation from a disease ecology point of view.

Finally, we hypothesize that the phenomenon of EBOV persistence and latency in bat species is a mechanism by which the virus can maintain itself long term in a highly-structured host population - i.e., high-density but geographically dispersed roosts. Younger bats dispersing to other colonies for mating, whilst persistently infected, may be the route of transmission between roosts. Furthermore, persistent infections within a roost may spark a new outbreak years later after sufficient immunologically-naïve individuals have been born. This then establishes a new cohort of persistently infected individuals. Persistence combined with periods of latency between episodes of replication and transmission is consistent with the phylogenetic patterns we observe between human outbreaks.

The reservoir composition and dynamics of Ebola virus have evaded characterisation since the virus was first isolated in the late 1970s. Our work here has used phylogenetic analyses to provide a rough sketch of the reservoir from its periodic spillover into humans. Filling in the details, the mechanisms behind latency, its implications for host dynamics, and its interaction with ecological processes like habitat fracturing, are crucial to understanding when and where EBOV will emerge next.

## Methods

### Data

To explore EBOV evolutionary rate variation in non-human hosts, we assembled a data set of genomes that span the known history of the virus. Most available EBOV genomes have been sampled from human cases. We have included one genome per outbreak, preferring those with precise dates of sampling. A list of sequences used is provided in Supplementary Table S2, including, where applicable, their GenBank or Pathoplexus accession numbers.

### Phylogenetic inference

Sequences were aligned using MAFFT (41) and refined by hand. Five prime and three prime untranslated regions were ignored in all phylogenetic analyses.

In each analysis, we used a separate HKY substitution model (42) for each of three partitions of the data: coding position one, coding position two, and a concatenation of coding position three and the intergenic regions. This was deemed to be the best partition scheme by IQ-TREE v2’s partition finder when compared to a partitioning scheme with separate partitions for the third coding position and intergenic regions. Maximum-likelihood phylogenetic trees were estimated using IQ-TREE v2.4.0(43). The likelihood ratio tests in the TipDate analysis (35) were performed using PAML (commit:ac9b97c8c35) (44, 45).

For the root-to-tip correlations in Figure 1, which exclude several clades, we first rooted the tree on the specified branch and then determined the location of the root on that branch based on most likely location from the TipDate analysis above. These calculations gave very similar results to those obtained from TempEst when run on trees containing just the tips included in the regressions (highlighted branches in Figure 1 A and B) (46).

Clade-level, maximum-likelihood evolutionary rate estimates were calculated using the strict-clock model proposed by Didelot *et al*. (47). Each clade was identified in and pruned out of the full maximum likelihood tree estimated by IQ-TREE v2 so that even clades with only 2 tips were rooted. We then estimated the maximum-likelihood evolutionary rate and time-scaled the branch lengths using a gamma distribution with mean equal to the variance as described in (47). Optimization was achieved using fmin (https://www.npmjs.com/package/fmin).

All Bayesian phylogenetic analyses were performed in BEAST X (48) using the BEAGLE high-performance computational library (49). For each analysis two chains were run for 500 million states and sub-sampled every 100 000 states with the first 50 000 states removed as burn-in. We employed an constant population coalescent tree prior with a log normal prior (μ:4,σ:1) ( 95% interval [8, 388]) on the population size. Preliminary analyses with an exponentially growing population did not exhibit a growth rate different from 0.

We placed an continuous-time Markov chain (CTMC) reference prior on the underlying evolutionary rate (50), with a slight modification. The CTMC prior is approximated as a gamma distribution with shape 0.5 and rate equal to the length of the tree, which results in an expectation of 0.5 mutations at each site over the entire tree. Because our tree contains periods during which no mutations accumulate, we updated the prior so that the rate was equal to the length of the replicating part of the tree which arrives at the same expectation.

We used an additional CTMC prior on the latent transition rate (see below for details). However, because latency is a branch specific event that ‘resets’ at each node and because we expect there to be some small amount of latency over the duration of the tree, we use the root height as the rate parameter. We place a uniform prior on the relative rate bias between entering and leaving the latent state. Sampling from these prior distributions results in a mean of 0.04 branches enjoying at least one period of latency with a 96% prior probability of no branches exibiting latency. We assessed convergence to the posterior distribution and proper mixing of all relevant parameters in Tracer 1.7 (51).

### Latent-state branch rate model

We model the evolutionary rate of EBOV according to a state-dependent rate model (52) where branches alternate between two possible states, replicating and latent, according to a CTMC.

Each branch transitions independently between states according to an infinitesimal rate matrix Q conditioned on starting and ending in the replicating state. This condition is necessitated by the fact that each node represents either a sampled active infection (external nodes) or an unsampled branching event (internal nodes). While in the replicating state, each branch undergoes evolution according to some underlying molecular clock rate model (e.g. strict clock, uncorrelated relaxed clock, etc.); the evolutionary rate is set to 0 when in the latent state. The overall evolutionary rate then along a branch is determined by its underlying rate (μ) and the amount of time spent in the replicating $\left({\text{t}}_{\text{r}}\right)$ and latent ${(\text{t}}_{\text{l}})$ states, where $\text{t}={\text{t}}_{\text{l}}+{\text{t}}_{\text{r}}$ is the total length of the branch in time.

Several approaches exist for estimating the amount of time a branch spends in each state. Cappello *et al*. explicitly sample the state history of each lineage as it transitions between replicating and non-replicating states in a seedbank model (53). While accurate, explicitly sampling complete transition histories is computationally expensive. Similarly, Lewinsohn *et al*. have developed an alternative state-dependent evolutionary rate model that marginalizes over the state history and approximates the evolutionary rate of each branch by using the expected time spent in each state (52). Such an approximation seems appropriate in cases where both states replicate at different rates. However, in our setting, where branches may not experience any latency the mean time spent latent is a poor representation of the possible dynamics.

We avoid sampling migration histories and instead estimate the proportion of time each branch spends in latency, integrated over all possible histories. Our approach is akin to recent approximations to the structured coalescent which also marginalize over histories (54–57). However, the model used here is simplified in that each branch’s migration history is independent of all others, which allows for an exact solution.

We are interested in estimating the phylogenetic posterior distribution described by

(1) $$\text{p}(\text{G},\Theta ,\Omega ,\kappa |\text{D})\propto \text{P}(\text{D}|\text{G},\Phi ,\kappa )\text{f}(\Phi |\Omega ){\text{f}}_{\text{T}}(\text{G}|\Theta )\pi (\Omega ,\Theta ,\kappa ),$$

where G is our phylogenetic tree with branch lengths in years, Θ represents the parameters governing the coalescent tree prior, Ω the parameters of the evolutionary model, and κ the substitution parameters. Moreover, P(D∣G,Φ,κ) is the observed sequence likelihood of an alignment D given the phylogenetic tree G, the collection of evolutionary rates (one for each branch) Φ, and the parameters governing the substitution model κ. Density ${\text{f}}_{\text{T}}(\text{G}|\Theta )$ is the tree prior with hyperparameters Θ,f(Φ∣Ω) the probability of each branch’s evolutionary rate according to the latent model described below, and π(Ω,Θ,κ) represents the prior over the remaining parameters.

In our model, Ω parametrizes the rate matrix underlying our latent-state CTMC and is comprised of two parameters, r and b, in addition to any parameters needed by the base evolutionary rate model – μ in the case of a strict clock. The rate matrix is defined as

(2) $$\text{Q}=\left[\begin{array}{cc} -\text{rb} & \text{rb}\\ \text{r}(1-\text{b}) & -\text{r}(1-\text{b}) \end{array}\right]$$

with the first state representing replication and the second latency.

Given that all nodes exist in the replicate state, the process on each branch is then independent of the others and so we write

(3) $$\text{f}\left(\Phi |\Omega \right)=\prod_{\text{i}=1}^{2\left(\text{n}-1\right)}\text{f}\left(\phi_{\text{i}}|\Omega \right),$$

where n is the number of tips in our tree, 2(n-1) is the number of branches and each branch has its own rate ϕ. We define each $\phi =\mu {\text{p}}_{\text{l}}$ as the product of a base evolutionary rate μ and the proportion of the branch length spent in latency ${\text{p}}_{\text{l}}$. Here, we use a strict clock model to assign the same base evolutionary rate to each branch, but more complicated models could be used. Thus,

(4) $$\text{f}\left(\phi |\Omega ,\text{t}\right)=\text{f}\left({\text{p}}_{\text{l}}|\Omega ,\text{t}\right),$$

where t is the length of the branch in time. We derive $f\left({\text{p}}_{\text{l}}|\Omega ,\text{t}\right)$ below.

We note that ${\text{p}}_{\text{l}}={\text{t}}_{\text{l}}/\text{t}$ and so

(5) $$\text{f}\left({\text{p}}_{\text{l}}|\Omega ,\text{t}\right)=\left\{\begin{array}{ll} \text{f}\left({\text{t}}_{\text{l}}|\Omega ,\text{t}\right)\cdot \text{t} & 0<{\text{p}}_{\text{l}}\leq 1,\\ \text{f}\left({\text{t}}_{\text{l}}|\Omega ,\text{t}\right) & {\text{p}}_{\text{l}}=0. \end{array}\right.$$

Let X(s) describe the state of a branch (i.e. rep, latent) at time s(0≤s≤t), and ${\text{t}}_{\text{l}}$ describe the occupation time spent in the latent state. Sericola and colleagues (58, 59) provide numerically stable algorithms for the cumulative distribution function

(6) $$\text{F}\left({\text{t}}_{\text{l}}\leq \text{s},\text{X}(\text{t})=\text{rep}|\text{X}(0)=\text{rep},\text{t},\Omega \right),\;\text{s}\in \left[0,\text{t}\right],$$

and probability distribution function

(7) $$\text{f}\left({\text{t}}_{\text{l}}=\text{s},\text{X}(\text{t})=\text{rep}|\text{X}(0)=\text{rep},\text{t},\Omega \right),\;\text{s}\in \left(0,\text{t}\right],$$

which are conditioned on the starting state being in the replicating state (X(0)=rep).

Here, we are interested in conditioning on both the starting (X(0)) and ending (X(t)) state and have

(8) $$\text{F}\left({\text{t}}_{\text{l}}\leq \text{s}|\text{X}(\text{t})=\text{rep},\text{X}(0)=\text{rep},\text{t},\Omega \right)=\frac{\text{F}\left({\text{t}}_{\text{l}}\leq \text{s},\text{X}(\text{t})=\text{rep}|\text{X}(0)=\text{rep},\text{t},\Omega \right)}{\text{f}(\text{X}(\text{t})=\text{rep}|\text{X}(0)=\text{rep})}$$

and

(9) $$\text{f}\left({\text{t}}_{\text{l}}=\text{s}|\text{X}(\text{t})=\text{rep},\text{X}(0)=\text{rep},\text{t},\Omega \right)=\frac{\text{f}\left({\text{t}}_{\text{l}}\leq \text{s},\text{X}(\text{t})=\text{rep}|\text{X}(0)=\text{rep},\text{t},\Omega \right)}{\text{f}(\text{X}(\text{t})=\text{rep}|\text{X}(0)=\text{rep})},$$

which we will write with a t subscript below (${\text{F}}_{\text{t}}\left({\text{t}}_{\text{l}}|\Omega ,\text{t}\right)$ and ${\text{f}}_{\text{t}}\left({\text{t}}_{\text{l}}|\Omega ,\text{t}\right)$) to denote they are functions of the total branch length t. The probability of never entering the latent state ${\text{t}}_{\text{l}}=0$ is given by F(0∣Ω,t). The probability distribution function of the proportion of time spent latent $\text{f}\left({\text{p}}_{\text{l}}|\Omega ,\text{t}\right)$ is then defined as

(10) $$\text{f}\left({\text{p}}_{\text{l}}|\Omega ,\text{t}\right)=\left\{\begin{array}{ll} \text{t}\cdot {\text{f}}_{\text{t}}\left({\text{p}}_{\text{l}}\cdot \text{t}|\Omega ,\text{t}\right) & 0<{\text{p}}_{\text{l}}\leq 1,\\ {\text{F}}_{\text{t}}(0|\Omega ,\text{t}) & {\text{p}}_{\text{l}}=0. \end{array}\right.$$

The latent-state model has been implemented in BEAST X (commit:edf73f) (48). In our analyses we place a uniform (0, 1) prior on b and modified CTMC prior with rate equal to the height of the root on on r.

## Supplementary Material

1

## Figures and Tables

**Figure 3:**
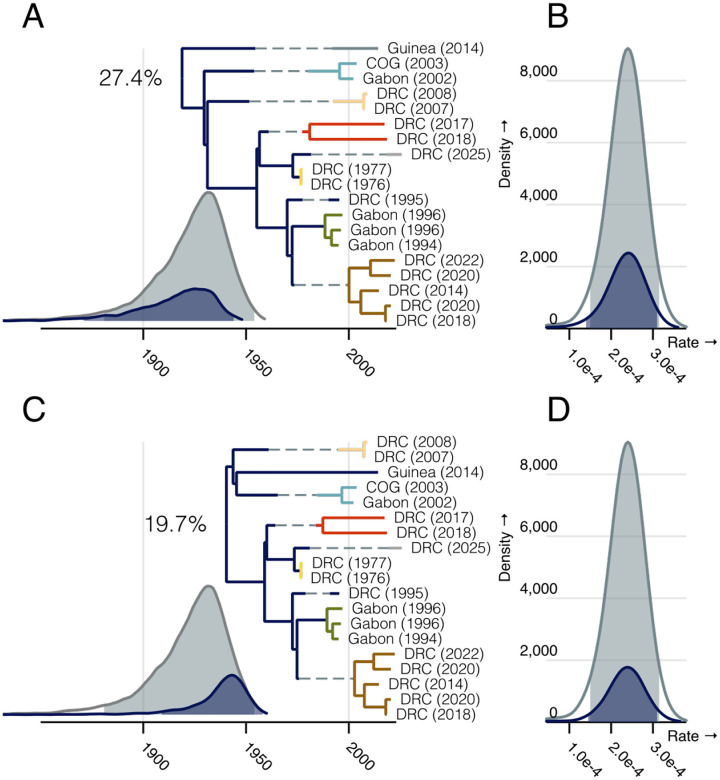
Posterior distributions of latency model parameters estimated for the full dataset partitioned by root position. A & C) MCC trees of the most common root placement (A) and the proposed root (C) (posterior probability noted near root). Clades are coloured as previously. The marginal posterior root age distribution is shown in grey with each root’s conditional contribution is highlighted in blue. The mean duration of latency (conditioned on there being at least one period of latency) is shown as a dashed line on branches with a posterior probability of any latency greater than 50%. B & D) The posterior distribution of the evolutionary rate during replication, again shaded by total analysis (grey) and conditioning on specific roots (blue). In all posterior distribution plots the shaded area represents the 95% HPD.

**Table 1:** Results of a TipDate analysis (35) for temporal signal in the 8 outbreaks highlighted in Figure 1B and subsequent partitions with local clocks placed on non-cluster branches subtending the remaining outbreak clusters.

Data set	Model	LL	ΔLL	df	p	Base rate	Root
Temporal set	DR	−29895.9	–	–	–	–	–
8 taxa	SRDT	−29898.0	−2.1	5	0.69	3.10×10^−4^	Proposed
	SRDT	−29900.3	−4.4	5	0.18	1.95×10^−4^	Midpoint
	SRDT	−29906.7	−10.9	5	< 0.001	7.56×10^−4^	1970s
	SR	−30036.9	−141.0	6	< 0.001	–	1970s
	SR	−30794.4	−898.6	6	< 0.001	–	Midpoint
	SR	−31454.9	−1559.0	6	< 0.001	–	Proposed
Classic temporal set	DR	−31462.7	–	–	–	–	–
11 taxa + Local clocks	localSRDT	−31466.6	−3.9	6	0.37	3.05×10^−4^	Proposed
	localSRDT	−31469.8	−7.1	6	0.03	2.02×10^−4^	Midpoint
	localSRDT	−31480.7	−18.0	6	< 0.001	7.48×10^−4^	1970s
	localSR	−31487.9	−25.2	7	< 0.001	–	Midpoint
	localSR	−31612.6	−149.9	7	< 0.001	–	1970s
	localSR	−38129.7	−6667.0	7	< 0.001	–	Proposed
Full data set	DR	−35710.2	–	–	–	–	–
19 taxa + Local clocks	localSRDT	−35714.7	−4.6	11	0.71	2.99×10^−4^	Proposed
	localSRDT	−35719.1	−8.9	11	0.11	2.19×10^−4^	Midpoint
	localSR	−35747.8	−37.7	12	< 0.001	–	Midpoint
	localSRDT	−35748.8	−38.6	11	< 0.001	6.99×10^−4^	1970s
	localSR	−35763.3	−53.1	12	< 0.001	–	Proposed
	localSR	−36017.2	−307.1	12	< 0.001	–	1970s

Model - the clock model used (DR: different rates, i.e. no clock; SR: single rate, i.e. unidentifiable clock; SRDT: single rate dated tips, i.e. identifiable clock; localSRDT - an SRDT model with local clock on between cluster branches not highlighted in Figure 1B), LL - Log likelihood, ΔLL - Log likelihood difference from the best model, df - degrees of freedom in a chi-squared test, p - p-value of a chisquared test, Base rate - the inferred evolutionary rate (where applicable), Root - the root position used in the analysis (see Supplemental Figure S1B)
