# Supplementary material for: Evidence of latency reshapes our understanding of Ebola virus reservoir dynamics
Source: bioRxiv. 2025 Oct 18:2025.10.17.683141. Preprint. [Version 1] doi: 10.1101/2025.10.17.683141 (PMC12632977; doi:10.1101/2025.10.17.683141)
Supplement: 1 [file NIHPP2025.10.17.683141V1-supplement-1.pdf]

bioRxiv preprint doi: https://doi.org/10.1101/2025.10.17.683141; this version posted October 18, 2025. The copyright holder for this preprint (which was not certified by peer review) is the author/funder, who has granted bioRxiv a license to display the preprint in perpetuity. It is made available under aCC-BY 4.0 International license.

Supplemental figures and text

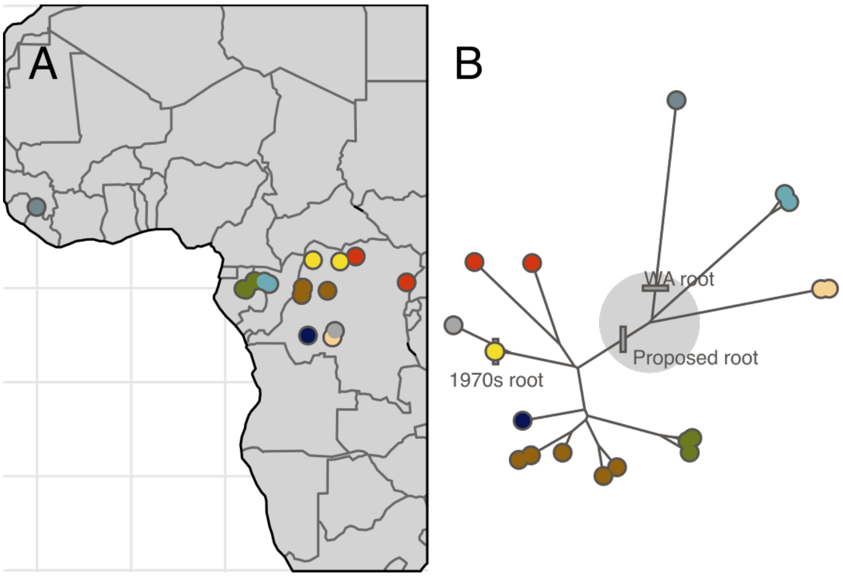

Figure S1: A map (A) and unrooted tree (B) representing the outbreaks included in this study. Common and proposed root placements are noted on the tree. The large grey sphere represents plausible root positions proposed by the latency rate model.

bioRxiv preprint doi: https://doi.org/10.1101/2025.10.17.683141; this version posted October 18, 2025. The copyright holder for this preprint (which was not certified by peer review) is the author/funder, who has granted bioRxiv a license to display the preprint in perpetuity. It is made available under aCC-BY 4.0 International license.

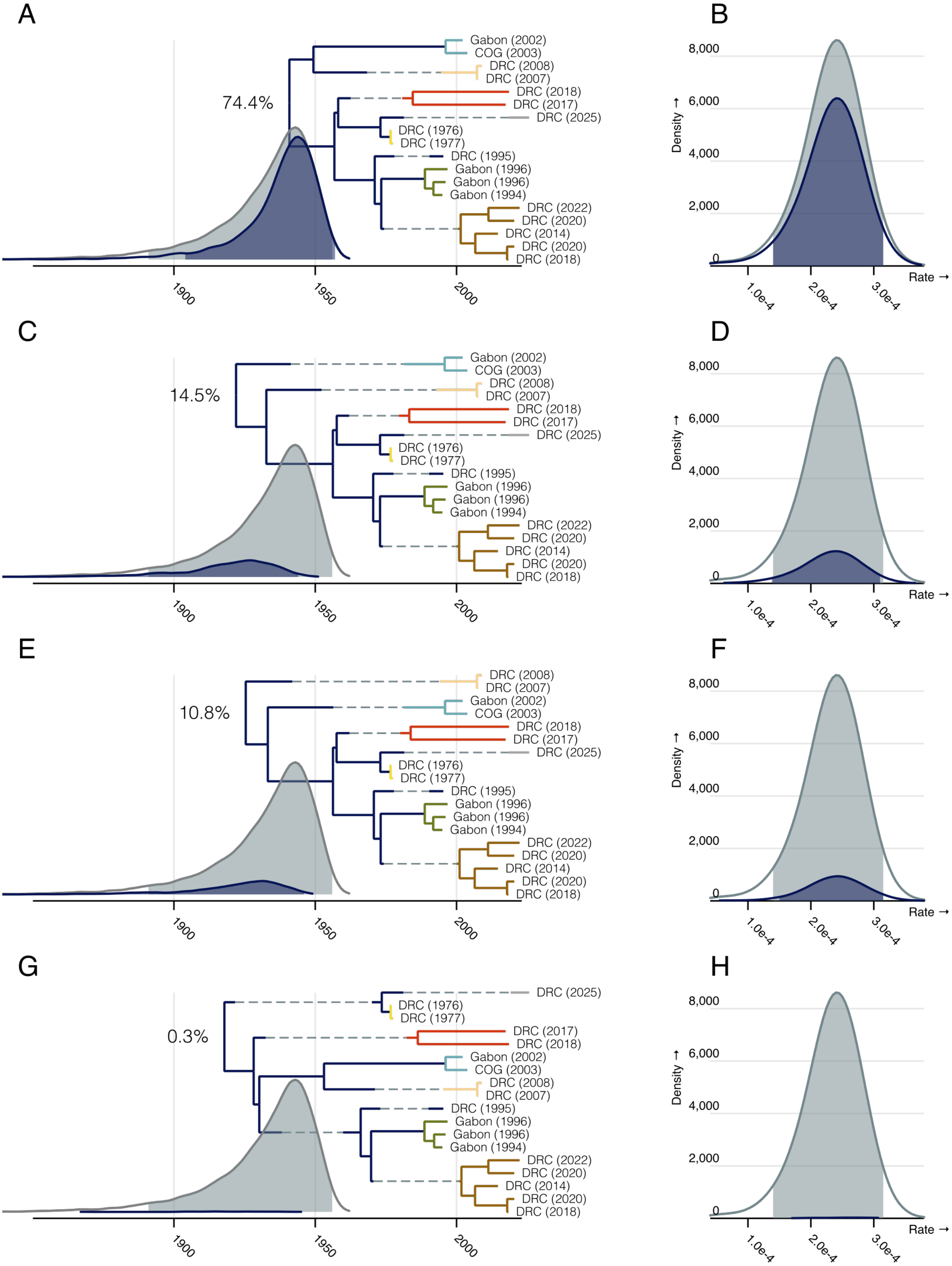

Figure S2: Posterior distributions of latency model parameters partitioned by root position for the four most common rootings in the Central African data set (i.e. no West African outbreak). A, C, E, & G) MCC trees of the most common root placements (posterior probabilities are noted near each root). Clades are coloured as previously. The marginal posterior root age distribution is shown in grey with each root's conditional contribution highlighted in blue. The mean duration of latency (conditioned on there being at least one period of latency) is shown as a dashed line on branches with a posterior probability of any latency greater than 50%. B, D, F, & H) The posterior distribution of the evolutionary rate during replication, again shaded by total analysis (grey) and conditioning on specific roots (blue). In all posterior distribution plots the shaded area represents the 95% HPD.

bioRxiv preprint doi: https://doi.org/10.1101/2025.10.17.683141; this version posted October 18, 2025. The copyright holder for this preprint (which was not certified by peer review) is the author/funder, who has granted bioRxiv a license to display the preprint in perpetuity. It is made available under aCC-BY 4.0 International license.

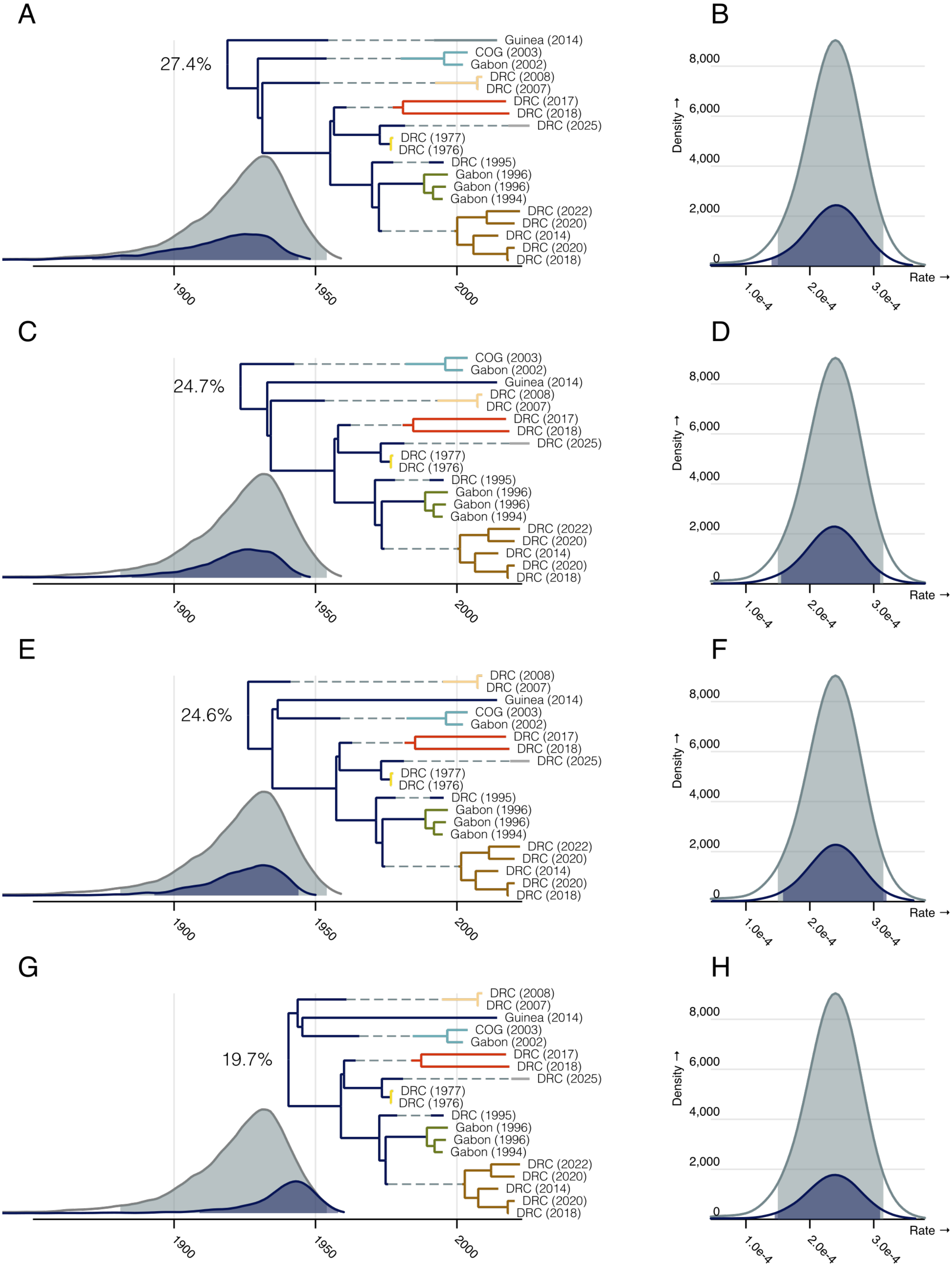

Figure S3: Posterior distributions of latency model parameters partitioned by root position for the four most common rootings in the full data set. A, C, E, & G) MCC trees of the most common root placements(posterior probabilities are noted near each root). Clades are colored as previously. The marginal posterior root age distribution is shown in grey with each root's conditional contribution highlighted in blue. The mean duration of latency (conditioned on there being at least one period of latency) is shown as a dashed line on branches with a posterior probability of any latency greater than 50%. B, D, F, & H) The posterior distribution of the evolutionary rate during replication, again shaded by total analysis (grey) and conditioning on specific roots (blue). In all posterior distribution plots the shaded area represents the 95% HPD.

bioRxiv preprint doi: https://doi.org/10.1101/2025.10.17.683141; this version posted October 18, 2025. The copyright holder for this preprint (which was not certified by peer review) is the author/funder, who has granted bioRxiv a license to display the preprint in perpetuity. It is made available under aCC-BY 4.0 International license.

outbreak	country	adm1	dates
Yambuku/1976	DRC	Mongala	Sep-Nov 1976
Sud-Ubangi/1977	DRC	Sud-Ubangi	Jun-77
Gabon/1994	GAB	Ogooue-Ivindo	Dec 1994-Feb 1995
Kwilu/1995	DRC	Kwilu	Jan-Jul 1995
Gabon/1996a	GAB	Ogooue-Ivindo	Jan–Apr 1996
Gabon/1996b	GAB	Ogooue-Ivindo	Jul-96
Gabon/2001	GAB	Ogooue-Ivindo	Oct 2001-Jul 2002
RoC/2003	COG	Cuvette Ouest	Oct-Dec 2003
Kasaï/2007	DRC	Kasai Occidental	May-Nov 2007
Kasaï/2008	DRC	Kasai Occidental	Dec 2008–Feb 2009
Makona/2013	GIN	Gueckedou	Dec 2013-Mar 2016
Tshuapa/2014	DRC	Tshuapa	Aug–Nov 2014
Bas-Uele/2017	DRC	Bas Uele	May-July 2017
Equateur/2018	DRC	Equateur	May-July 2018
Kivu/2018	DRC	Nord Kivu - Ituri - South Kiev	Aug 2018-June 2020
Equateur/2020	DRC	Equateur	June - Nov 2020
Mbandaka/2022	DRC	Equateur	April- July 2022
Kasaï/2025	DRC	Kasai Occidental	August - ongoing

Table S1: Recorded EBOV outbreaks used in this study. amd1 - Administrative Region 1 of the outbreak.

accession	country	outbreak	date	authors
KR063671	DRC	Yambuku/1976	1976-10-01	Das,S.R., Shabman,R., Halpin,R.A., Lin,X., Ransier,A., Fedorova,N., Tsitrin,T., Puri,V., Stockwell,T., Amedeo,P., Bishop,B., Gupta,N., Katzel,D., Schobel,S., Shrivastava,S., Alfson,K., Wentworth,D.E. and Griffiths,A.
KC242791	DRC	Sud-Ubangi/1977	1977-06	Carroll,S.A., Towner,J.S., Sealy,T.K., McMullan,L.K., Khristova,M.L., Burt,F.J., Swanepoel,R., Rollin,P.E. and Nichol,S.T.
KC242792	GAB	Gabon/1994	1994-12-27	Carroll,S.A., Towner,J.S., Sealy,T.K., McMullan,L.K., Khristova,M.L., Burt,F.J., Swanepoel,R., Rollin,P.E. and Nichol,S.T.
KU182905	DRC	Kwilu/1995	1995-05-04	Das,S.R., Shabman,R., Halpin,R.A., Akopov,A., Fedorova,N., Puri,V., Stockwell,T., Amedeo,P., Bishop,B., Katzel,D., Schobel,S., Shrivastava,S., Brasel,T., Yun,N., Paessler,S., Dowling,W. and Barrett,A.
KC242793	GAB	Gabon/1996a	1996-02	Carroll,S.A., Towner,J.S., Sealy,T.K., McMullan,L.K., Khristova,M.L., Burt,F.J., Swanepoel,R., Rollin,P.E. and Nichol,S.T.
KC242798	GAB	Gabon/1996b	1996-10-27	Carroll,S.A., Towner,J.S., Sealy,T.K., McMullan,L.K., Khristova,M.L., Burt,F.J., Swanepoel,R., Rollin,P.E. and Nichol,S.T.
KC242800	GAB	Gabon/2001	2002-02-23	Carroll,S.A., Towner,J.S., Sealy,T.K., McMullan,L.K., Khristova,M.L., Burt,F.J., Swanepoel,R., Rollin,P.E. and Nichol,S.T.
KF113529	COG	RoC/2003	2003-10	Chiu,C.Y., Naccache,S.N., Fair,J.N., Schneider,B.S. and Grard,G.
HQ613403	DRC	Kasaï/2007	2007-08-31	Grard,G., Biek,R., Muyembe-Tamfum,J.-J., Formenty,P., Paweska,J. and Leroy,E.
HQ613402	DRC	Kasaï/2008	2008-12-31	Grard,G., Biek,R., Muyembe-Tamfum,J.-J., Formenty,P., Paweska,J. and Leroy,E.
KJ660347	GIN	Makona/2013	2014-03-20	Baize,S., Pannetier,D., Oestereich,L., Rieger,T., Koivogui,L., Magassouba,N., Soropogui,B., Sow,M.S., Keita,S., De Clerck,H., Tiffany,A., Dominguez,G., Loua,M., Traore,A., Kolie,M., Malano,E.R., Heleze,E., Bocquin,A., Mely,S., Raoul,H., Caro,V., Cadar,D., Gabriel,M., Pahlmann,M., Tappe,D., Schmidt-Chanasit,J., Impouma,B., Diallo,A.K., Formenty,P., Van Herp,M. and Gunther,S.
KP271018	DRC	Tshuapa/2014	2014-08-20	Naccache,S.N., Mbala,P., Ngay,I., Makuwa,M., Mulembakani,P., Muyembe,J.J., Schneider,B.S. and Chiu,C.Y.
MH613311	DRC	Bas-Uele/2017	2017-05-07	Nsio,J., Kapetshi,J., Makiala,S., Formenty,P., Raymond,F., Tshapenda,G., Boucher,N., Corbeil,J., Okitandjate,A., Mbuyi,G., Kiyele,M., Mondonge,V., Kikoo,M.J., Van Herp,M., Rollin,P., Barboza,P., Muyembe Muzinga,B., Kalenga,O.I., Ahuka,S., Fausther-Bovendo,H., Kebela Ilunga,B., Kobinger,G.P. and Muyembe,J.-J.T.
MH733477	DRC	Equateur/2018	2018-05-10	Mbala,P., Pratt,C., Wiley,M.R., Makiala-Mandanda,S., Aziza,A., Di Paola,N., Diagne,M.M., Chitty,J.A., Diop,M., Ayouba,A., Vidal,N., Faye,O., Karhemere,S., Aruna,A., Nsio,J., Mulangu,F., Mukadi,D., Mukadi,P., Kombe,J., Mulumba,A., Duraffour,S., Likofata,J., Pukuta,E., Minogue,T., Sozhamannan,S., Gross,S., Schroth,G., Delaporte,E., Sanchez-Lockhart,M., Peeters,M., Muyembe,J.-J., Alpha Sall,A., Palacios,G. and Ahuka-Mundeke,S.

bioRxiv preprint doi: https://doi.org/10.1101/2025.10.17.683141; this version posted October 18, 2025. The copyright holder for this preprint (which was not certified by peer review) is the author/funder, who has granted bioRxiv a license to display the preprint in perpetuity. It is made available under aCC-BY 4.0 International license.

accession	country	outbreak	date	authors
MK007330	DRC	Kivu/2018	2018-07-28	Mbala-Kingebeni,P., Aziza,A., Di Paola,N., Wiley,M.R., Makiala-Mandanda,S., Caviness,K., Pratt,C.B., Prieto,K., Chitty,J.A., Larson,P., Ayouba,A., Vidal,N., Karhemere,S., Diop,M., Diagne,M.M., Faye,M., Faye,O., Aruna,A., Nsio,J., Mulanga,F., Mukadi,D., Mukadi,P., Kombe,J., Mulumba,A., Duraffour,S., Likofata,J., Pukuta,E., Gonzalez,J., Bartlett,M.L., Sozhamannan,S., Gross,S., Schroth,G., Kuhn,J., Delaporte,E., Sanchez-Lockhart,M., Alpha Sall,A., Muyembe,J.-J., Peeters,M., Palacios,G. and Ahuka-Mundeke,S.
OR084849	DRC	Equateur/2020	2020-05-31	Kinganda-Lusamaki,E., Whitmer,S., Lokilo-Lofiko,E., Amuri-Aziza,A., Muyembe-Mawete,F., Makangara-Cigolo,J.C., Makaya,G., Mbuyi,F., Whitesell,A., Kallay,R., Choi,M., Pratt,C., Mukadi-Bamuleka,D., Kavunga-Membo,H., Matondo-Kuamfumu,M., Mambu-Mbika,F., Ekila-Ifinji,R., Shoemaker,T., Stewart,M., Eng,J., Rajan,A., Soke,G.N., Fonjungo,P.N., Otshudiema,J.O., Folefack,G.L.T., Pukuta-Simbu,E., Talundzic,E., Shedroff,E., Bokete,J.L., Legand,A., Formenty,P., Mores,C.N., Porzucek,A.J., Tritsch,S.R., Kombe,J., Tshapenda,G., Mulangu,F., Ayouba,A., Delaporte,E., Peeters,M., Wiley,M.R., Montgomery,J.M., Klena,J.D., Muyembe-Tamfum,J.-J., Ahuka-Mundeke,S. and Mbala-Kingebeni,P.
OR084846	DRC	Equateur/2020	2020-06-12	Kinganda-Lusamaki,E., Whitmer,S., Lokilo-Lofiko,E., Amuri-Aziza,A., Muyembe-Mawete,F., Makangara-Cigolo,J.C., Makaya,G., Mbuyi,F., Whitesell,A., Kallay,R., Choi,M., Pratt,C., Mukadi-Bamuleka,D., Kavunga-Membo,H., Matondo-Kuamfumu,M., Mambu-Mbika,F., Ekila-Ifinji,R., Shoemaker,T., Stewart,M., Eng,J., Rajan,A., Soke,G.N., Fonjungo,P.N., Otshudiema,J.O., Folefack,G.L.T., Pukuta-Simbu,E., Talundzic,E., Shedroff,E., Bokete,J.L., Legand,A., Formenty,P., Mores,C.N., Porzucek,A.J., Tritsch,S.R., Kombe,J., Tshapenda,G., Mulangu,F., Ayouba,A., Delaporte,E., Peeters,M., Wiley,M.R., Montgomery,J.M., Klena,J.D., Muyembe-Tamfum,J.-J., Ahuka-Mundeke,S. and Mbala-Kingebeni,P.
PP_003V5NQ	DRC	Equateur/2022	2022-04-25	Placide Mbala-Kingebeni, Jean-Jacques Muyembe, Steve Ahuka-Mundeke, Hugo Kavunga, Daniel Mukadi, Elisabeth Pukuta, Eddy Kinganda Lusamaki, Amuri Aziza, Jean Claude Makangara, Emmanuel Lokilo, Franck Edidi, Junior Bula Bula, Raphael Lumembe, Gabriel Kabamba, Fabrice Mambu, Joel Montgomery, Peter Fonjungo, Norbert Soke, Jacques Likofata, Vital Mondonge, Gervais Folefack, Prosper Djiguimde, John Otshudiema, Deby Mukendi, Dieudonné Mwamba, John Kombe, Sofonias Tessema, Didi Bofaka, Andrew Rambaut, Mike Wiley, Catherine Pratt

bioRxiv preprint doi: https://doi.org/10.1101/2025.10.17.683141; this version posted October 18, 2025. The copyright holder for this preprint (which was not certified by peer review) is the author/funder, who has granted bioRxiv a license to display the preprint in perpetuity. It is made available under aCC-BY 4.0 International license.

accession	country	outbreak	date	authors
PP_003RXHG	DRC	Kasaï/2025	2025-09-01	Adrienne Amuri-Aziza, Gradi Luakanda - Ndelemo, Jean-Claude Makangara-Cigolo, Prince Akil-Bandali, Princesse Paku-Tshambu, Sam Wilkinson, Louis Tshulo, Josh Quick, Andre Citenga, Chloe Muswamba-Kayembe, Olga Ntumba-Tshitenge, Emmanuel Lokilo-Lofiko, Fiston Cikaya-Kankolongo, Ola Rilia, Servet Kimbonza, Elisabeth Pukuta, Daniel Mukadi, Christian Ngandu, Mathias Mossoko, Gabriel Kabamba -Lungenyi, Raphael Lumembe-Numbi, Elzedeck Mabika-Bope, Patrick Mukadi, Catherine Pratt, Dieudonne Mwamba, Nick Loman, Andrew Rambaut, Eddy Kinganda-Lusamaki, Tony Wawina-Bokalanga, Dieudonne Mumba - Ngoyi, Jean-Jacques Muyembe-Tamfum, Steve Ahuka-Mundeke Placide Mbala-Kingebeni

Table S2: A list of the genomes used in this study with outbreak and author list.

bioRxiv preprint doi: https://doi.org/10.1101/2025.10.17.683141; this version posted October 18, 2025. The copyright holder for this preprint (which was not certified by peer review) is the author/funder, who has granted bioRxiv a license to display the preprint in perpetuity. It is made available under aCC-BY 4.0 International license.
